# Cerebral Regulation in Different Maximal Aerobic Exercise Modes

**DOI:** 10.3389/fphys.2016.00253

**Published:** 2016-07-05

**Authors:** Flávio O. Pires, Carlos A. S. dos Anjos, Roberto J. M. Covolan, Fabiano A. Pinheiro, Alan St Clair Gibson, Timothy D. Noakes, Fernando H. Magalhães, Carlos Ugrinowitsch

**Affiliations:** ^1^Exercise Psychophysiology Research Group, School of Arts, Sciences, and Humanities, University of São PauloSão Paulo, Brazil; ^2^Department of Sport, School of Physical Education and Sport, University of São PauloSão Paulo, Brazil; ^3^Neurophysics Group, Gleb Wataghin Physics Institute, University of CampinasCampinas, Brazil; ^4^School of Medicine, University of the Free StateBloemfontein, South Africa; ^5^Department of Human Biology, Sports Science Institute of South Africa, University of Cape TownCape Town, South Africa

**Keywords:** near-infrared spectroscopy, brain oxygenation, exercise tolerance, central fatigue, peripheral muscle fatigue

## Abstract

We investigated cerebral responses, simultaneously with peripheral and ratings of perceived exertion (RPE) responses, during different VO_2MAX_-matched aerobic exercise modes. Nine cyclists (VO_2MAX_ of 57.5 ± 6.2 ml·kg^−1^·min^−1^) performed a maximal, controlled-pace incremental test (MIT) and a self-paced 4 km time trial (TT_4km_). Measures of cerebral (COX) and muscular (MOX) oxygenation were assessed throughout the exercises by changes in oxy- (O_2_Hb) and deoxy-hemoglobin (HHb) concentrations over the prefrontal cortex (PFC) and vastus lateralis (VL) muscle, respectively. Primary motor cortex (PMC) electroencephalography (EEG), VL, and rectus femoris EMG were also assessed throughout the trials, together with power output and cardiopulmonary responses. The RPE was obtained at regular intervals. Similar motor output (EMG and power output) occurred from 70% of the duration in MIT and TT_4km_, despite the greater motor output, muscle deoxygenation (↓ MOX) and cardiopulmonary responses in TT_4km_ before that point. Regarding cerebral responses, there was a lower COX (↓ O_2_Hb concentrations in PFC) at 20, 30, 40, 50 and 60%, but greater at 100% of the TT_4km_ duration when compared to MIT. The alpha wave EEG in PMC remained constant throughout the exercise modes, with greater values in TT_4km_. The RPE was maximal at the endpoint in both exercises, but it increased slower in TT_4km_ than in MIT. Results showed that similar motor output and effort tolerance were attained at the closing stages of different VO_2MAX_-matched aerobic exercises, although the different disturbance until that point. Regardless of different COX responses during most of the exercises duration, activation in PMC was preserved throughout the exercises, suggesting that these responses may be part of a centrally-coordinated exercise regulation.

## Introduction

Studies originally suggested that cerebral oxygenation (COX) measured at the prefrontal cortex (PFC) is involved with the capacity to perform maximal aerobic exercises (Nielsen et al., 1999; Rupp and Perrey, 2008). Results obtained during controlled-pace, maximal incremental exercise test (MIT) at sea level indicated an increase in COX up to ≈80% of the peak power output (W_PEAK_), but a decrease from this intensity toward the exercise endpoint (Rupp and Perrey, 2008; Subudhi et al., 2008). This reduction in COX during the last 20% of the MIT, after the second ventilatory threshold (VT_2_), matched a pronounced increase in muscle recruitment as measured by electromyography (EMG), suggesting that the ability to increase motor output at maximal levels was related to the deoxygenation in PFC areas (Rupp and Perrey, 2008; Perrey, 2009). In addition, this may have suggested that activation of the primary motor cortex (PMC) was not impaired, despite the COX drop in PFC areas. Therefore, at least at sea level, reductions in COX may reflect the ability of the central nervous system (CNS) to maximally increase muscle recruitment and power output at the closing stages of a MIT.

Using a different maximal exercise mode, Billaut et al. (2010) showed a similar reduction in COX at the end-spurt of a 5 km self-paced running time trial (i.e., the last 10% of the trial), which time-matched an increase in EMG. Furthermore, COX also increased during the first half of the exercise, as observed in MIT (Billaut et al., 2010). Hence, evidences provided by independent studies suggested that reductions in COX measured at PFC matched, but did not limit the increase in motor output (i.e., EMG and power output) at the closing stages of maximal controlled-pace (Rupp and Perrey, 2008) or self-paced exercise (Billaut et al., 2010). They further suggested that deoxygenation in PFC did not impair the activation in motor cortex areas during maximal motor output in these exercises.

Responses of COX may be part of a process of exercise regulation. Robertson and Marino (2016) have suggested that the PFC has a vital role for the exercise regulation and tolerance, as it integrates afferents from peripheral organs and muscles into emotional messages used to guide the decision-making in exercise. Homeostatic disturbances during exercise are integrated into stimulus motivationally significant in PFC regions, guiding the decision to continue or stop the exercise (Craig, 2002; Robertson and Marino, 2016). This regulation possibly tax the PFC areas (i.e., metabolic cost), as the interpretation of the benefit of activating motor cortex areas while tolerating unpleasant sensations triggered by homeostatic disruption is processed by PFC areas (Meeusen et al., 2016). In this sense, the capacity to perform maximal aerobic exercises could be linked to the ability of the PFC to articulate emotional messages while preserving the PMC activity, even under great metabolic disruption. In fact, independent studies using electroencephalography technique (EEG) have shown that activation in PMC can be preserved during MIT (Brümmer et al., 2011; Robertson and Marino, 2015), despite the pronounced PFC deoxygenation and homeostatic disruption that occur after the VT_2_, during the last 20% of the trial (Rupp and Perrey, 2008). However, if the same response would be found in maximal self-paced exercises is yet to be verified. A recent study found a 6–12% reduction in maximal voluntary activation superimposed by transcranial magnetic stimulation (TMS) after different self-paced cycling trials, thereby suggesting that some impairment in PMC activation occurred during the exercise (Thomas et al., 2015). Therefore, an experimental setup including PFC oxygenation and PMC activation measures during maximal aerobic exercises may be insightful.

The process of exercise regulation may depend on the exercise mode under consideration, as the ability to tolerate homeostatic disruption seems to be different between controlled-pace and self-paced exercises (Lander et al., 2009; Robertson and Marino, 2016). A previous study found greater homeostatic disruption in submaximal controlled-pace exercise, as the core temperature, blood lactate concentrations and EMG were higher than in a power-matched, submaximal self-paced exercise. Accordingly, individuals perceived the controlled-pace exercise as more challenging, given the higher ratings of perceived exertion (RPE) in this exercise mode. However, it remains allusive if the different homeostatic disturbances between controlled-pace and self-paced exercise would affect the ability of the PFC to integrate these responses into emotional messages, thus impairing the PMC activation necessary to increase the motor output at maximal levels.

Therefore, the present study explored cerebral responses (PFC oxygenation and PMC activation) to maximal, controlled-pace and self-paced exercise. We further measured peripheral [muscle oxygenation (MOX)], cardiopulmonary and RPE responses to these exercises. We hypothesized that regardless of reductions in COX at the closing stages of the exercises, PMC activation would be preserved in both the exercise modes, even though the motor output was maximal. However, we did not known about differences in cerebral responses to these exercises.

## Materials and methods

### Participants and experimental design

Nine trained male road cyclists (32.9 ± 7.3 years old, body mass of 75.9 ± 9.0 kg, height of 175.7 ± 5.9 cm, and body fat of 10.5 ± 5.2%), experienced (≥3 years) in short distance cycling time trials, volunteered to participate in this study. They were non-smokers with no neuromuscular or cardiopulmonary disorders. The procedures were explained to the participants before they were asked to sign an informed consent form. The experimental protocol conformed to the Declaration of Helsinki, being previously approved by the University of São Paulo's Ethics Committee (0023.0.342.000-10).

It is important to note that a previous study compared controlled-pace and self-paced exercises by using a submaximal power-matched exercise design (Lander et al., 2009). Instead, in the present study individuals were required to perform maximal motor output in both the exercise modes, so that we used a VO_2MAX_-matched exercise design to accomplish this goal. In this regard, whole body exercises that elicit VO_2MAX_ (i.e., maximum oxygen uptake) values have been interpreted as maximal aerobic exercises, according to the traditional maximal aerobic exercise performance paradigm (Bassett and Howley, 2000; Levine, 2008). Thus, we utilized a MIT routinely used to assess VO_2MAX_ as maximal controlled-pace exercise, because this exercise mode requires the maintenance of a target pedal cadence while the workload is increased. In contrast, a 4 km cycling time trial (TT_4km_) was chosen as maximal self-paced aerobic exercise, as this exercise mode provides VO_2_ values similar to VO_2MAX_ values obtained in MIT (Mauger et al., 2009; Williams et al., 2012). However, individuals are free to pace themselves during this exercise mode, while the distance is completed within the fastest possible time.

The cyclists were habituated to perform laboratory MIT and TT_4km_, as they took part in previous study that used these physical exercise tests. Thus, the experimental design comprised the following sequential visits to the laboratory: (1) first visit to measure body mass, height, pectoral, abdominal, and thigh skinfold thickness (Harpenden®, West Sussex, UK), and to familiarize them with the experimental procedures (bicycle, cycle-simulator, Borg's scale, cerebral, and muscular measures); (2) second visit to perform a controlled-pace MIT; (3) third visit to perform a self-paced TT_4km_. Important, previous studies have provided evidences for TT_4km_ reliability in trained and untrained individuals (Mauger et al., 2009; Williams et al., 2012). All tests were performed at the same time of the day, in a laboratory with temperature (21⋅C) and humidity (50–60%) controlled. Visits 2 and 3 were interspersed by ≈7 days, and the entire study lasted for ≈15 days. The cyclists were encouraged to maintain the training schedule (intensity and volume) throughout the study and to avoid exercises, alcohol or stimulant beverages for the 24 h before the tests.

Throughout the MIT and TT_4km_, COX, and MOX were continuously measured through near-infrared spectroscopy (NIRS) technology, while PMC and muscle activation were continuously assessed through EEG and EMG, respectively. Furthermore, cardiopulmonary variables were continuously sampled, while RPE was obtained at regular intervals.

### Exercise protocols

After being prepared for experimental procedures, cyclists were accommodated on the bicycle; then they closed their eyes and remained in absolute rest for 2 min for baseline measurements. Immediately after the baseline assessments, a standard 7 min warm-up, consisting of a 5 min self-paced exercise and a 2 min controlled-pace exercise (cycling at 100 W with pedal cadence of 80 rpm), was performed before the MIT and TT_4km_ exercises. The 2 min controlled-pace exercise warm-up was used to normalize the exercise EMG data. The MIT was initiated immediately after the standard warm-up, while cyclists were still cycling at 100 W (80 rpm) during the controlled-pace warm-up. The power output was increased 25 W·min^−1^ until exhaustion, while the pedal cadence was maintained at 80 rpm. Exhaustion was determined when the pedal cadence dropped below the 80 rpm, despite strong verbal encouragement. Similarly, after the standard warm-up, cyclists immediately began the TT_4km_ while receiving verbal encouragement to complete the trial as fast as possible. Elapsed time and distance were available throughout the test, so that cyclists were free to pace themselves in the TT_4km_.

### Instruments

The controlled-pace MIT and the self-paced TT_4km_ were performed on a speed bicycle (Giant®, Thousand Oaks, CA, USA) attached to a cycle-simulator (Racer Mate®, Computrainer, Seattle, WA, EUA), and equipped with a crank (SRM®, PowerControl 7, Jülich, Köln, Germany) that provided power output (W) data at a 2 Hz frequency. The cycle-simulator was calibrated before every test, according to the manufacturer's instructions. The validity and reliability of these instruments have been reported elsewhere (Duc et al., 2007; Peveler, 2013).

Changes in COX and MOX were assessed through alterations in oxy-hemoglobin (O_2_Hb) and deoxy-hemoglobin (HHb) concentrations throughout the experimental set-up, via NIRS (CW6- TechEn®, Milford, MA, USA) at a sampling rate of 25 Hz. This system monitors the tissue absorption via optical fibers optodes with light sources and detectors. The COX was monitored with optodes placed over the prefrontal lobe at the Fp1 position, according to the international 10–20 system (Subudhi et al., 2008; Billaut et al., 2010; Santos-Concejero et al., 2015). The MOX was monitored over the right vastus lateralis (VL) muscle, the optodes were positioned at ~15 cm proximal and 5 cm lateral from the superior border of the patella (Subudhi et al., 2009); the adipose thickness was ≤5 mm at this point. Adhesive tape was used to fix the optodes into a specific plastic holder, projected with a 4.5 cm inter-optode distance to reduce artifacts during the exercise. Briefly, this inter-optode distance was chosen because it is within the range suggested to obtaining reliable NIRS measures in muscle and brain. For example, a 4.5 cm inter-optode distance has been suggested to be unaffected by changes in scalp blood flow or adipose tissue thickness (Hamaoka et al., 2007; Ekkekakis, 2009). Moreover, a modified Beer-Lambert law, based on optical densities from two continuous wavelengths of 690 and 830 nm, calculated the μmol changes in tissue O_2_Hb and HHb, respectively (Perrey, 2008; Ekkekakis, 2009).

The activation in PMC was monitored throughout the experimental set-up by an EEG unit (NicoletOne V32®, Viasys Healthcare Inc., Madison, WI, USA). After measuring circumferences, frontal and sagittal planes to ensure the electrode placement according to the EEG international 10–20 system, active electrode (Ag-AgCl) was fixed with medical strips and conductive gel at the Cz position (referenced to mastoid), after exfoliation and cleaning. This position was selected to represent the cortical area responsible for motor commands to lower limbs muscles, according to the human motor homunculus (Nielsen et al., 2001; Nybo and Nielsen, 2001; Goodall et al., 2014). The electrode-skin impedance was kept <5 KΩ throughout the experimental protocol, and the electrode position was confirmed after the exercise. The surface signal was amplified (gain of 1000) and sampled at 2 kHz, using a 1–30 Hz band pass filter. In order to reduce artifacts, EEG unit's cables were fixed on the individuals' trunk, and cyclists were further oriented to maintain upper limbs as steady as possible during exercise (Thompson et al., 2008). A researcher experienced with EEG records visually inspected the data for noise, so that data with excessive noise was excluded from the EEG analysis (*n* = 2).

The EMG of the VL and rectus femoris (RF) muscles was monitored throughout the standard warm-up and exercise (Delsys®, Chicago, IL, USA). A pair of surface EMG electrodes (impedance <5kΩ, inter-electrode distance of 2 cm) was placed over the muscle belly according to the probable muscle fiber orientation of these muscles, after cleaning, shaving and exfoliation of the skin. A surgical pen ensured the same electrode placement between the testing sessions. The EMG signal was amplified (gain of 1000) and sampled at 2 kHz.

Cardiopulmonary responses including VO_2_ (ml·kg^−1^·min^−1^), ventilation (VE; L·min^−1^) and end-expiratory fraction of CO_2_ (FeCO_2_ expressed as %) were assessed using an open-system gas analyzer (Cosmed®, Quark PT, Albano Laziale, Rome, Italy), which was calibrated before each test with ambient air and gases of known composition (20.9% O_2_ and 5% CO_2_). The turbine flowmeter was calibrated with a 3 L syringe (Quinton Instruments®, Milwaukee, WI, USA). The cyclists wore a mask (Hans Rudolph®, Lenexa, KS, USA) connected to the gas analyzer to obtain breath-by-breath measurements of gaseous exchange throughout the experimental set-up. In addition, the heart rate (HR) was obtained throughout the experimental set-up (2 Hz frequency) by a cardio belt (Suunto®, Finland).

The cyclists were instructed to rate their perceived exertion (RPE) according to the 6–20 Borg's scale (Borg, 1982), at the end of every stage in the MIT, and at the end of every 0.5 km in the TT_4km_. They were oriented to consider breathlessness, cardiac work, muscular strain, and body temperature when reporting the RPE (Hampson et al., 2001).

### Data analysis

In order to analyze time and exercise mode effects, experimental data were plotted as a function of paired percentages of the total exercise duration. Thus, after data processing as described below, values of power output, COX, MOX, EEG, EMG, and cardiopulmonary responses were averaged over the last 10 s of every 10% of the total exercise duration. In contrast, irregular time intervals of RPE measures implicated in non-paired percentages between MIT and TT_4km_, thus the RPE slope was used to compare the exercise modes.

#### Power output

Power output data were re-sampled to 1 Hz to remove extreme values, before averaging values at 10% intervals. The time to exhaustion and the time to complete the 4 km were further used as a performance parameter in MIT and TT_4km_, respectively. In order to characterize the cyclists, the W_PEAK_ achieved in the MIT and TT_4km_, calculated as the mean of the highest 30 s of the trial, was recorded.

#### NIRS data

The raw data were filtered using a 0.4 Hz low pass-band filter (HomER, http://www.nmr.mgh.harvard.edu/PMI/resources/homer/home.htm), and subsequently resampled to 1 Hz. Then, exercise NIRS data were expressed relative to the last 30 s of the baseline (Δμmol), before averaging values at 10% intervals. During this baseline period participants were completely calm, with their eyes closed and without voluntary movements. The O_2_Hb, HHb, and total hemoglobin concentrations (THb = O_2_Hb + HHb) were indexes of COX and MOX (Subudhi et al., 2008; Billaut et al., 2010; Santos-Concejero et al., 2015).

#### EEG data

The exercise EEG data were normalized to the signal recorded in the last 30 s of the baseline. In order to analyze the frequency domain data, power spectrum density was estimated by the Welch periodogram of detrended data, resulting in a resolution of 0.2 Hz. The area under the power spectrum curve at 10% exercise intervals was calculated for the alpha wave (α), between 8 and 13 Hz. Briefly, we have used the alpha bandwidth at Cz position to indicate PMC activation, as alpha wave levels in motor cortex areas may reflect an increased number of neurons coherently activated (Pfurtscheller and Lopes da Silva, 1999). The greater inhibited neurons-to-disinhibited neurons relationship in motor cortices suggests higher activation, reflecting the facilitation of sensory stimuli derived from PFC areas during subjective awareness (Craig, 2002; Uusberg et al., 2013). Thus, an increased alpha level during exercise may indicate a cooperative or synchronized behavior of a large number of activated neurons, therefore reflecting activation (von Stein and Sarnthein, 2000; Robertson and Marino, 2015). In addition, this frequency band has turned out to be sensitive to motor activity with less EMG activity contamination (Hilty et al., 2011).

#### EMG data

The EMG signal was initially filtered with a hardware band-pass filter set at 20 and 500 Hz, thereafter the exercise EMG data were normalized as a function of the EMG recorded during the standard controlled-pace warm-up (Knutson et al., 1994; Ball and Scurr, 2013). The root mean square (RMS) calculated at every 10% of the exercise duration, provided an index of activation in VL and RF muscles.

#### Cardiopulmonary data

Breath-by-breath data were filtered using moving averages, and values ≥3 SD from the local mean (the 5-breath moving average) were substituted by the local mean. Thereafter, a cubic spline interpolation provided data at every 1 s interval (DiMenna et al., 2008), before averaging the last 10 s of every 10% of the exercise duration. Important, based on the study design's rationale, we identified the VO_2MAX_ in MIT. The VO_2MAX_ was determined by the plateau criterion, an increase in VO_2_ of ≤150 ml·min^−1^ (Taylor et al., 1955). Alternatively, VO_2MAX_ was determined when RER > 1.10, peak HR > 90% of the age-estimated HR, and RPE > 18, if a VO_2_ plateau was not detected (Shephard, 1984). In addition, three evaluators identified the VT_2_ visually, thus making possible comparisons with previous studies which reported cerebral responses relative to VT_2_ (Rupp and Perrey, 2008; Robertson and Marino, 2015). The second increase in the VE/VO_2_ relationship during MIT determined the VT_2_ intensity (Meyer et al., 2005).

#### RPE data

The slope derived from linear regression of the RPE-time of exercise relationship was calculated to indicate how RPE changed over the different exercise modes.

### Statistics

Gaussian distribution was initially checked through Shapiro–Wilk's test. Dependent variables with regard to motor output (power output and VL and RF muscles EMG), cerebral (COX and EEG α wave), and peripheral (MOX and cardiopulmonary variables) responses were compared within and between the different exercise modes through a number of true mixed models, having time and mode of exercise as fixed factors and cyclists as random factor (Ugrinowitsch et al., 2004). When *F*-values were significant, multiple comparisons were performed through Bonferroni's test; rather than fixed factors, we were interested in the time-by-exercise mode interaction. The RPE slope during both the exercise modes was compared through a paired Student's *t*-test. All the significant p values (*p* < 0.05) attained a power ≥0.98. Furthermore, effect sizes (ES) for main and interaction effects were calculated (expressed as Cohen's d) and interpreted as small (*d* ≤ 0.20), moderate (0.20 < d < 0.80) and large (*d* ≥ 0.80). Results were reported as mean and standard deviation (± SD).

## Results

### Cyclists' characterization

Most cyclists (*n* = 8) achieved VO_2MAX_ according to the plateau criteria, the exception was one cyclist who showed a VO_2_ plateau between 150 and 200 ml·min^−1^, so that alternative criteria were used to determine his VO_2MAX_. They achieved a VO_2MAX_ of 57.5 ± 6.2 ml·kg^−1^·min^−1^, a W_PEAK_ of 368.4 ± 19.3 W, and a VT_2_ of 282.2 ± 23.6 W (76.6 ± 6.4% W_PEAK_) during MIT. Regarding the TT_4km_, cyclists used a U-shape pacing strategy, thus reaching a W_PEAK_ of 391.9 ± 78.7 W within the first 10% of trial. The mean power output during TT_4km_ was 316.8 ± 62.0 W. The MIT was completed within 699 ± 67 s, and the TT_4km_ within 359 ± 17 s.

### Motor output

**Figure 1:**
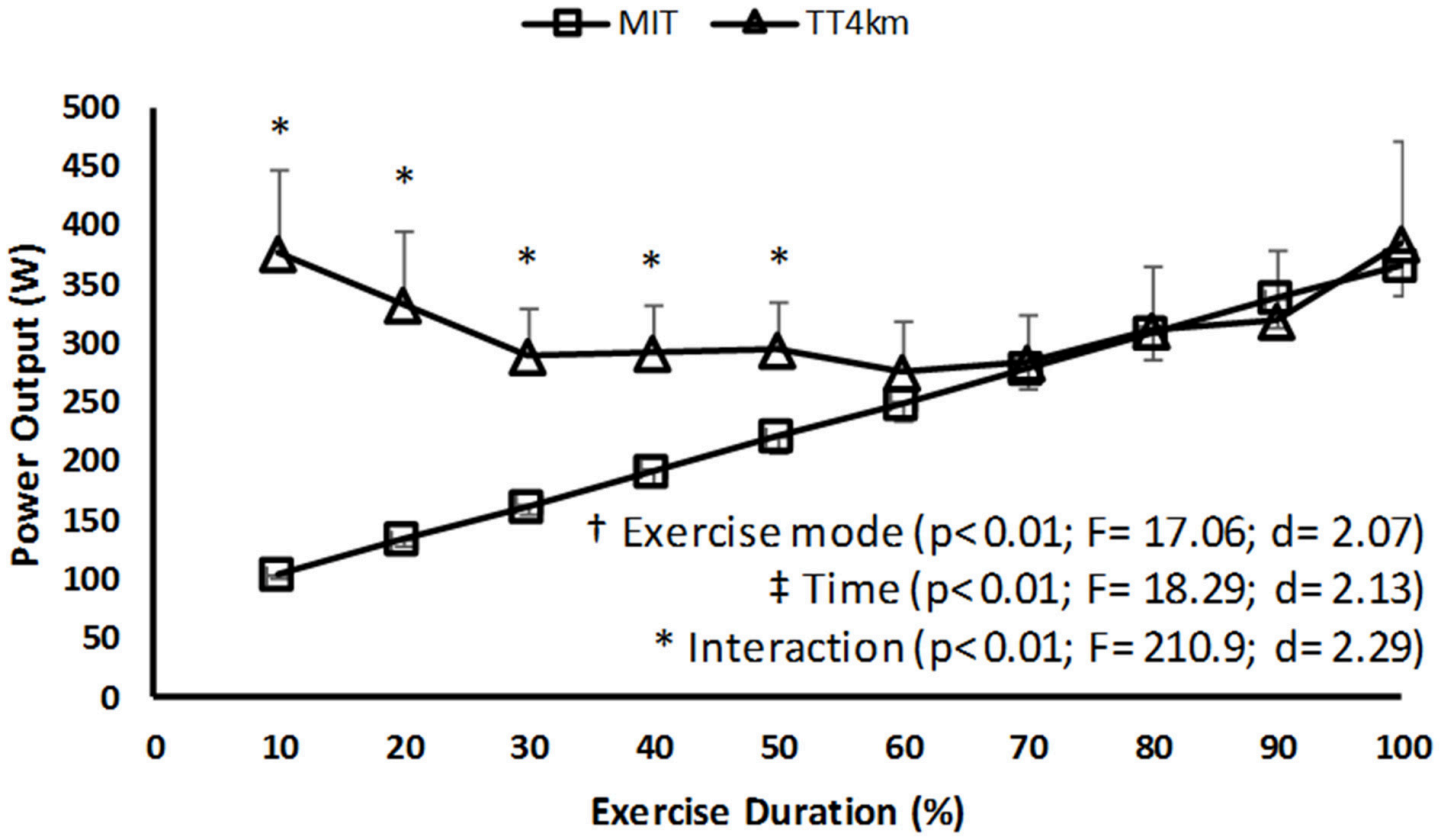
**Power output during maximal controlled-pace (MIT) and self-paced (TT_**4km**_) exercise**. Symbols indicate exercise mode (†) and time (‡) main effects, as well as time-by-exercise mode interaction effects (^*^).

A time (*P* < 0.001) main effect was observed, so that the power output changed over time for both the exercise modes. Additionally, an exercise mode (*P* < 0.01) main effect reveled greater values in TT_4km_ than in MIT. A time-by-exercise mode interaction effect (*P* < 0.01) revealed higher power output values in TT_4km_ than MIT, from 10 to 50% of the exercise duration (Figure 1).

**Figure 2:**
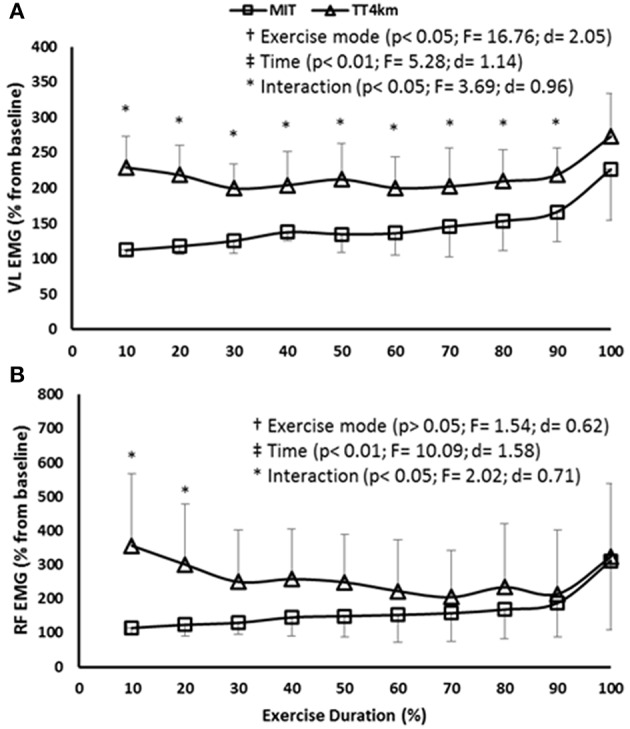
**(A,B)** EMG of the vastus lateralis (VL) and rectus femoris (RF) muscle during maximal controlled-pace (MIT) and self-paced (TT_4km_) exercise. Symbols indicate exercise mode (†) and time (‡) main effects, as well as time-by-exercise mode interaction effects (^*^). Panels **(A,B)** depict VL and RF EMG.

Regarding EMG responses, a time main effect (*P* < 0.001) was found in VL and RF muscles, but only VL muscle showed an exercise mode main effect (*P* < 0.05). In addition, there was a time-by-exercise mode interaction effect in VL and RF muscles (*P* < 0.05). As shown by Figures 2A,B, overall results were a greater muscle activation in TT_4km_, mainly during the first half of the trial.

### Cerebral responses

**Figure 3:**
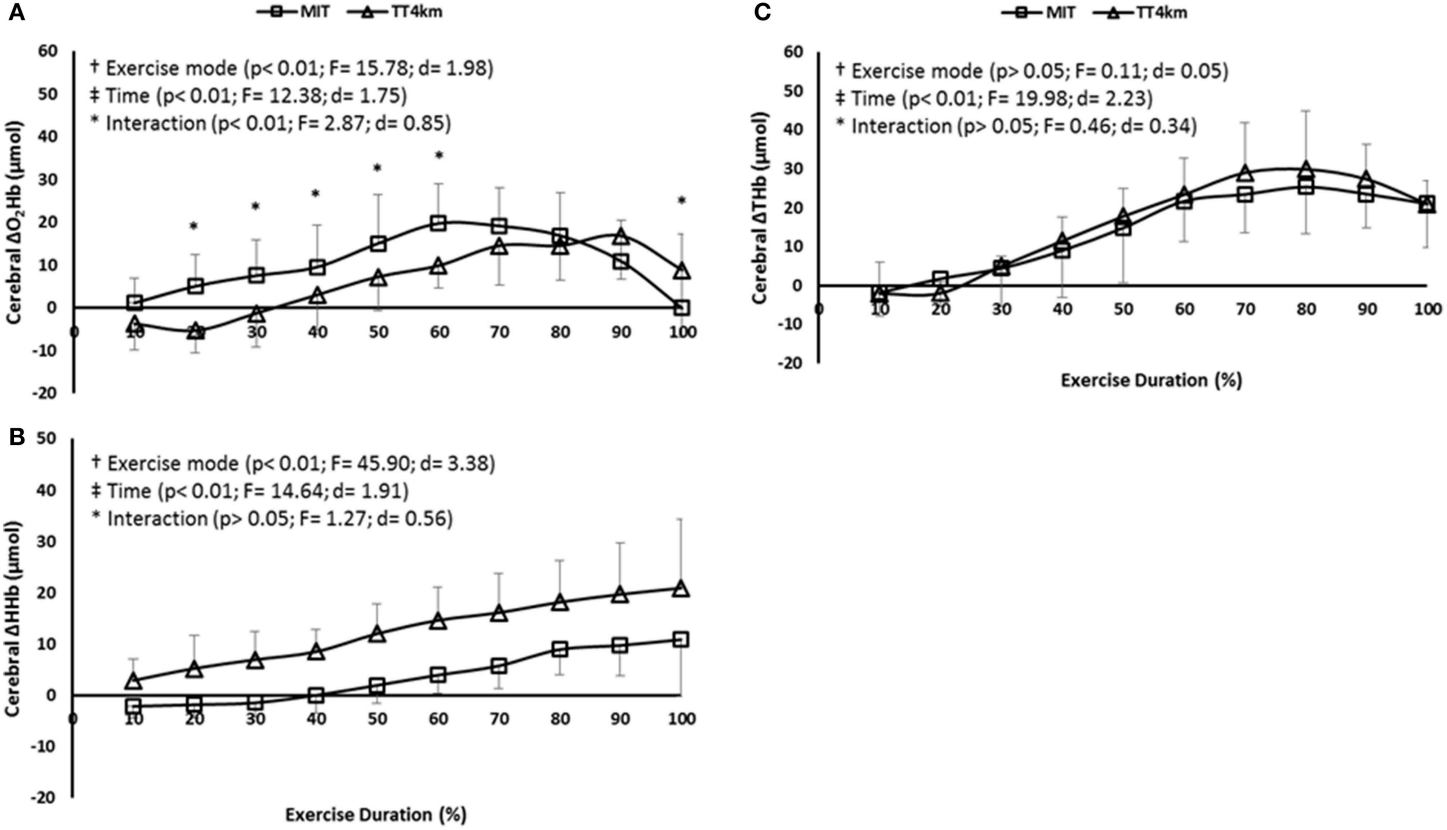
**(A–C)** Cerebral oxygenation (COX) measured at the prefrontal cortex (PFC) during maximal controlled-pace (MIT) and self-paced (TT_4km_) exercise. Symbols indicate exercise mode (†) and time (‡) main effects, as well as time-by-exercise mode interaction effects (^*^). Panels **(A–C)** depict Δ[O_2_Hb], Δ[HHb], and Δ[THb].

Regarding COX responses, a time and an exercise mode main effect were observed for Δ[O_2_Hb] (*P* < 0.001) and Δ[HHb] (*P* < 0.001), but only a main time effect was found in Δ[THb] (*P* < 0.001). Thus, oxygenation responses at PFC increased up to ≈70% of both MIT and TT_4km_, and decrease afterwards. A time-by-exercise mode interaction effect was observed for Δ[O_2_Hb] (*P* < 0.01), thus when compared to MIT, Δ[O_2_Hb] was lower in TT_4km_ than MIT at 20, 30, 40, 50, and 60%, but higher at 100% of the exercise duration (Figures 3A–C).

**Figure 4:**
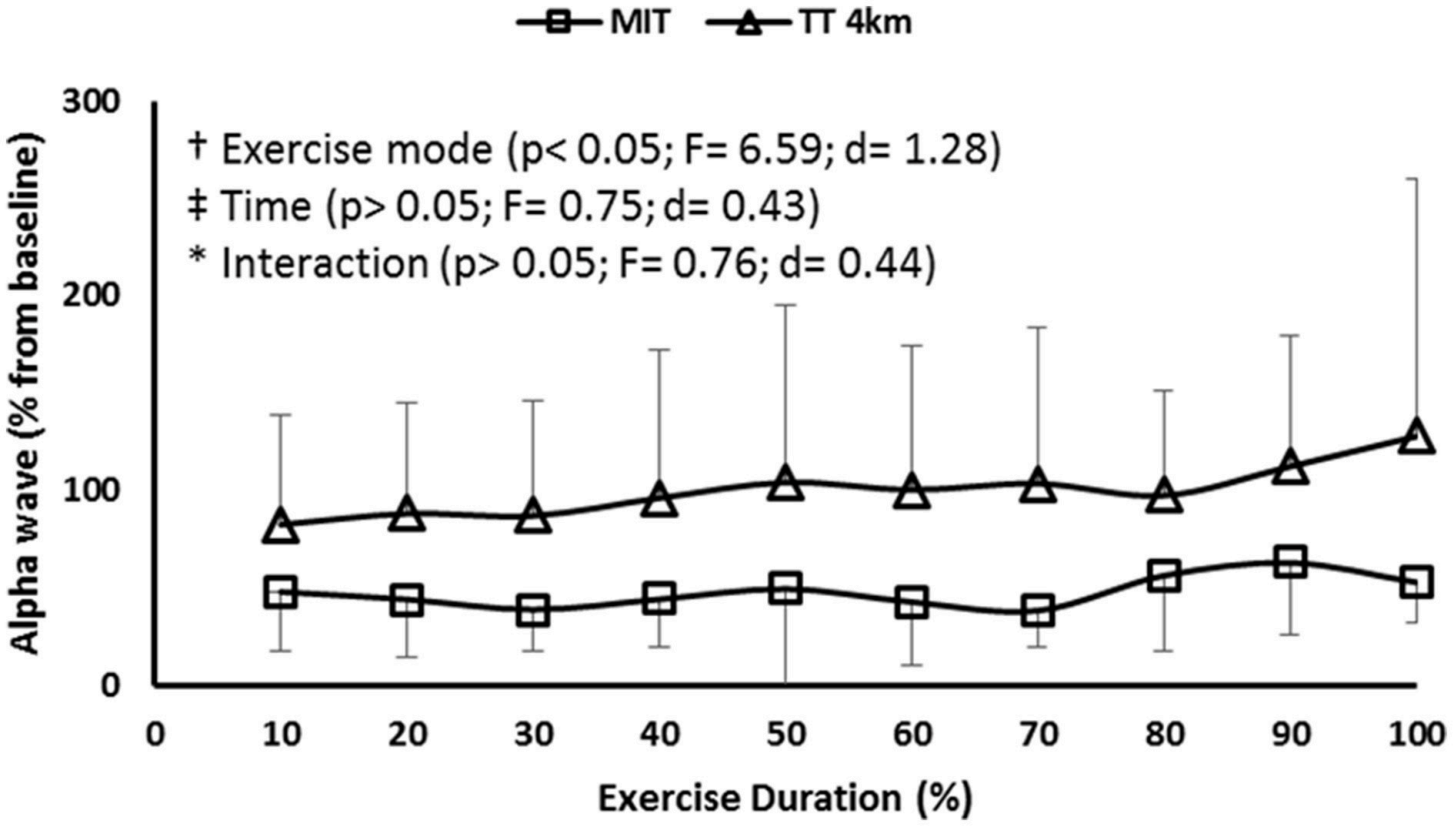
**EEG α wave measured at the Cz position during maximal controlled-pace (MIT) and self-paced (TT_**4km**_) exercise**. Symbols indicate exercise mode (†) and time (‡) main effects, as well as time-by-exercise mode interaction effects (^*^).

The activation in PMC was maintained in both MIT and TT_4km_, as neither time nor time-by-exercise mode interaction effect was observed in EEG α band at the Cz position. However, an exercise mode main effect (*P* < 0.01) showed a higher activation in TT_4km_ (Figure 4).

### Peripheral responses

**Figure 5:**
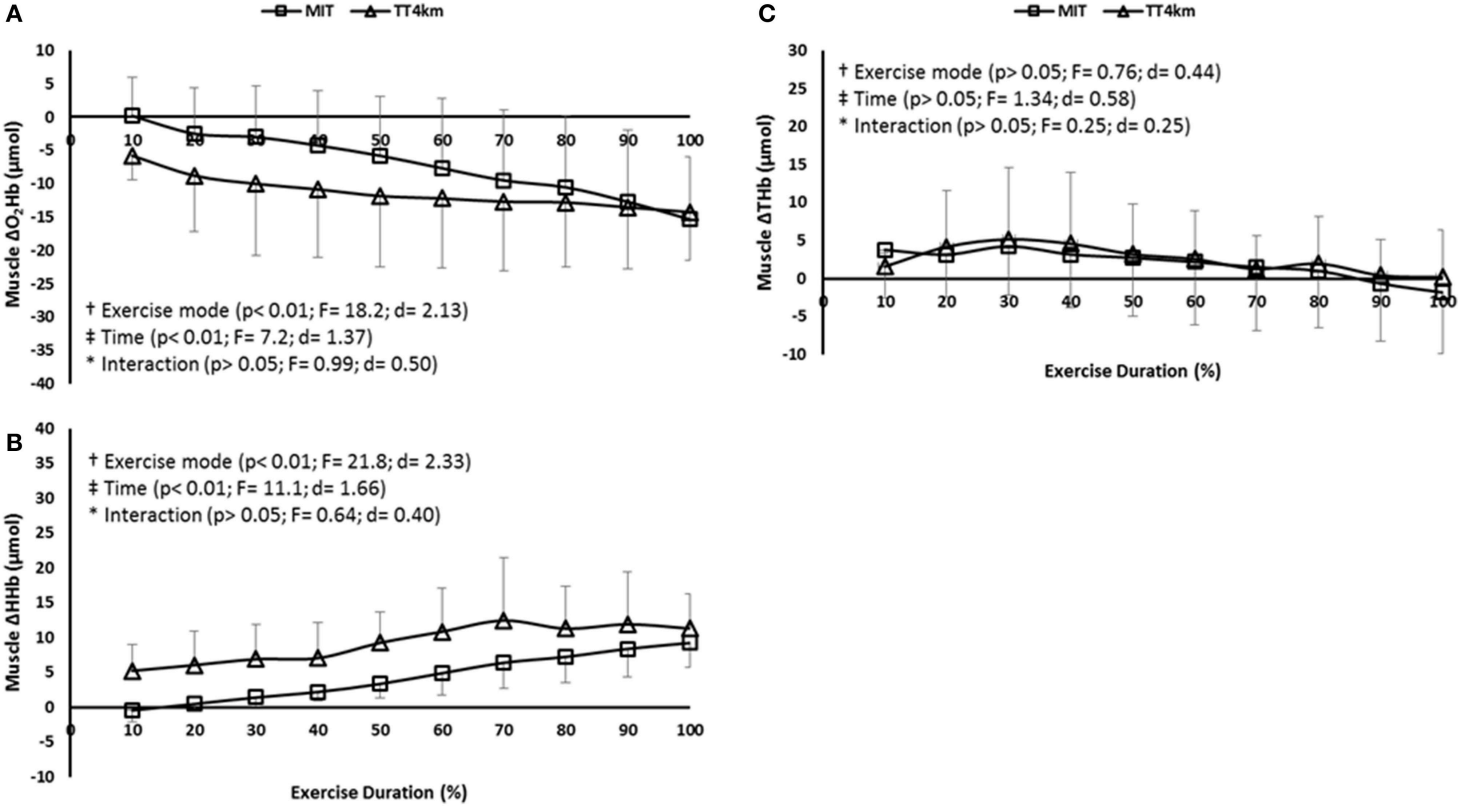
**(A–C)** Muscular oxygenation (MOX) measured in vastus lateralis muscle (VL) during maximal controlled-pace (MIT) and self-paced (TT_4km_) exercise. Symbols indicate exercise mode (†) and time (‡) main effects, as well as time-by-exercise mode interaction effects (^*^). Panels **(A–C)** depict Δ[O_2_Hb], Δ[HHb], and Δ[THb].

MOX responses (Δ[O_2_Hb] and Δ[HHb], but not Δ[THb]) decreased up to ≈50% of the MIT and TT_4km_ (time main effect; *P* < 0.001), with lower values for this latter exercise mode (exercise mode main effect; *P* < 0.001). No time-by-exercise mode interaction effect was observed for MOX indices (Figures 5A–C).

**Figure 6:**
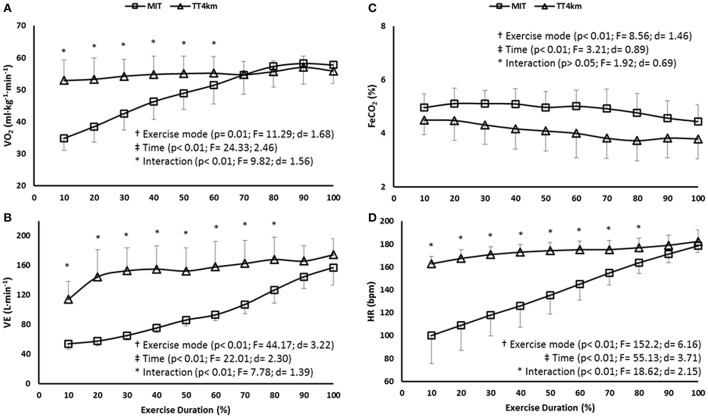
**(A–D)** Cardiopulmonary responses during maximal controlled-pace (MIT) and self-paced (TT_4km_) exercise. Symbols indicate exercise mode (†) and time (‡) main effects, and time-by-exercise mode interaction effects (^*^). Panels **(A–D)** depict VO_2_, VE, FeCO_2_, and HR.

Cardiopulmonary responses increased throughout the exercise modes (time main effect; *P* < 0.001), and values in the TT_4km_ were systematically greater than in MIT (exercise mode main effect; *P* < 0.01), mainly during the first half of the trials (time-by-exercise mode interaction effect; *P* < 0.01; Figures 6A–D).

### Perceived exertion

**Figure 7:**
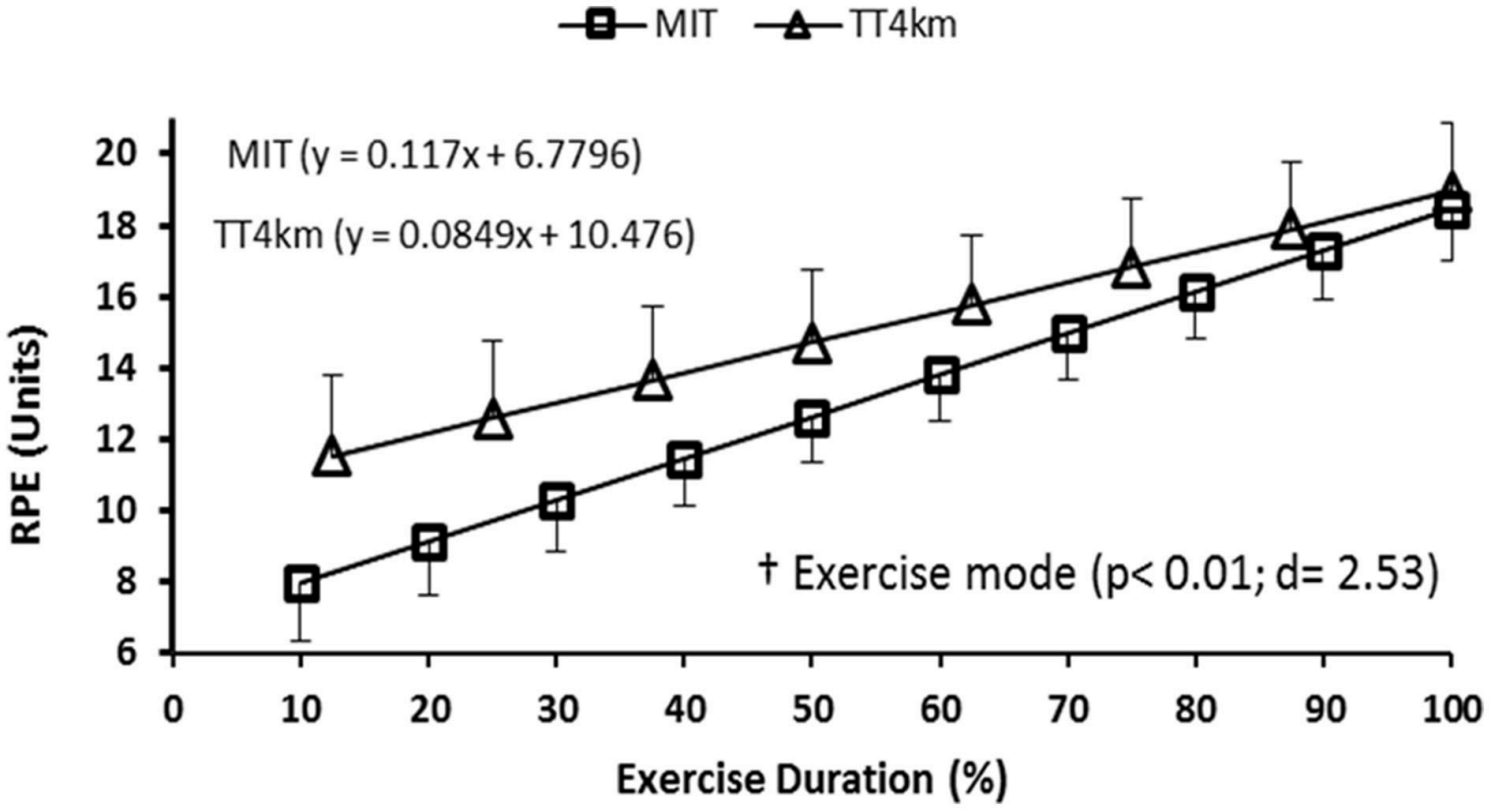
**RPE responses during maximal controlled-pace (MIT) and self-paced (TT_**4km**_) exercise**. Symbol indicates exercise mode main effect (†) for RPE slope.

The different exercise modes induced different effort sensations, as there was a greater RPE slope (*P* < 0.01) in MIT than in TT_4km_ (Figure 7).

## Discussion

We hypothesized that COX reductions at the closing stages of the exercises would not impair the PMC activation, thus allowing an increasing muscle recruitment and power output (i.e., motor output) at maximal levels in both the exercise modes. The present study showed that different aerobic exercise modes induced similar, maximal motor output during the last 50% of the VO_2MAX_-matched trials, regardless of COX reductions (Δ[O_2_Hb]) from 70% of both exercises. The different motor output performed before that point implicated in distinct COX responses during most of the exercises, however the PMC activation was preserved throughout the exercises. Interestingly, cyclists perceived the self-paced TT_4km_ more tolerable than MIT, even though the greater motor output and intensified physiological responses during the trial.

COX responses in both exercise modes may be partially linked to a systemic network response initiated by an increasing motor output. For example, elevations in motor output (mainly after the VT_2_) can lead to elevations in cardiopulmonary responses such as hyperventilation, probably due to the greater III/IV afferent fibers-induced central command to respiratory and vascular systems (Amann et al., 2010, 2011). As a result, hyperventilation-induced hypocapnia would drive cerebral responses toward vasoconstriction and diminished blood flow, thus affecting Δ[O_2_Hb] and Δ[THb] concentrations (Nielsen et al., 1999). In fact, reductions in Δ[O_2_Hb] and Δ[THb] concentrations over time (main time effects) matched an elevated motor output, hyperventilation and lowered FeCO_2_ levels (Figure 6C).

The different maximal exercise modes caused different PFC deoxygenation, since TT_4km_ induced lower Δ[O_2_Hb] concentrations between 20 and 60% of the total duration (but greater at the endpoint) when compared to MIT. It has been suggested that COX responses to exercise may reflect O_2_ tissue extraction rather than O_2_ tissue delivery (Millet et al., 2012), thus the Δ[O_2_Hb] concentrations relative to Δ[THb] concentrations may indicate the local O_2_ tissue extraction (González-Alonso et al., 2004). Therefore, when compared to MIT, the lower Δ[O_2_Hb] relative to Δ[THb] concentrations during most of the TT_4km_ may have indicated that the maximal self-paced TT_4km_ required greater O_2_ tissue extraction than MIT.

This different O_2_ tissue extraction between the exercise modes may be part of a centrally-coordinated exercise regulation, suggesting a distinct participation of the PFC between MIT and TT_4km_. The PFC projects to motor cortex areas, and may regulate motor output when integrating sensory afferent information to guide the decision-making during exercise (Ridderinkhof et al., 2004; Robertson and Marino, 2015). Thus, it is possible that the self-paced TT_4km_ has required a higher PFC activation to integrate the sensory afferents from skeletal muscles and cardiopulmonary system, thus resulting in a greater O_2_ tissue extraction (↓ Δ[O_2_Hb]) in this area. In fact, the greater muscle deoxygenation (MOX ↓) and cardiopulmonary response (VO_2_, VE, FeCO_2_, and HR) during most of the self-paced TT_4km_ could suggest that there was a higher III/IV muscle afferents toward interoceptive PFC areas in this exercise mode (Craig, 2002; Amann et al., 2011; Meeusen et al., 2016).

Importantly, PMC activation was maintained throughout the exercise modes, regardless of different PFC deoxygenation patterns. These results are in agreement with recent findings showing that PMC activation can be preserved throughout a MIT, even at intensities above the VT_2_ (Robertson and Marino, 2015), an exercise intensity that elicits pronounced changes in COX, EMG, and cardiopulmonary responses (Rupp and Perrey, 2008). Our results provide support to the hypothesis that motor cortex activation may be preferentially preserved during strenuous exercises, in contrast to PFC areas (Subudhi et al., 2009; Robertson and Marino, 2015). In addition, results of EEG α wave further indicated greater PMC activation in TT_4km_ than in MIT, agreeing with the elevated motor output (i.e., EMG and power output) during most of this exercise mode. This could suggest a greater coherence of neural populations activation in PMC regions during TT_4km_, perhaps reflecting a higher facilitation of sensory stimuli derived from the PFC in this self-paced exercise (Uusberg et al., 2013).

Interestingly, although the higher motor output and exaggerated PFC deoxygenation during the first half of the TT_4km_, cyclists perceived this self-paced exercise as more tolerable than MIT, as RPE increased slower until the attainment of maximal values. Such a response may be linked to the nature of the exercise mode under consideration (Robertson and Marino, 2016). Different of controlled-pace exercises, during which individuals are forced to match a power output predetermined by the experimenter, self-paced exercises allow individuals to pace themselves according to feedback and feedforward control mechanisms (Marino et al., 2011; Noakes, 2012). In this exercise mode the CNS is allowed to regulate the recruitment/de-recruitment of motor units, so that the rate of increase in physiological disturbance would be reduced due to an alleviated muscle recruitment (Marino et al., 2011; Noakes, 2012). Actually, when comparing a power-matched, submaximal self-paced exercise with a forced-pace exercise, previous study observed a lower physiological disturbance which was associated with a less challenging perception in this former exercise mode (Lander et al., 2009).

The fact that the physiological disturbance was exacerbated in the self-paced exercise, as suggested by the lower MOX and higher cardiopulmonary responses to the TT_4km_, does not necessarily contradicts those previous results in submaximal exercises (Lander et al., 2009), since the exercises were performed at maximal motor output levels in the present study. Perhaps the continuous muscle recruitment/de-recruitment regulation via feedback and feedforward commands during the self-paced TT_4km_ has allowed a more appropriate sensory cues integration into interoceptive PFC areas (Robertson and Marino, 2016), thus taxing the PFC with a higher metabolic cost (lower Δ[O_2_Hb] relative to Δ[THb] concentrations). However, as indicated by RPE responses, the self-selection of the exercise intensity made this exercise mode more tolerable than the controlled-pace MIT, possibly as a result of a greater affective response during this exercise mode. In fact, a recent meta-analysis concluded that exercises with self-selected intensity promote greater affective response than imposed ones (Oliveira et al., 2015). Then, considering the fact that affect and RPE responses to exercise may share the same conscious mental processing (Ramalho Oliveira et al., 2015), perhaps the greater tolerance in maximal self-paced exercise could be a result of the greater affective response when self-selecting the pace of exercise.

### Aspects of the study's design and limitations

We have assumed the VO_2MAX_ as the upper limit for maximal aerobic exercise performance (Bassett and Howley, 2000; Levine, 2008), so that we used VO_2MAX_-matched exercises while investigating cerebral responses to maximal controlled-pace and self-paced exercise. This approach ascribed rationale to the study, as we measured PFC oxygenation and PMC activation (together with peripheral variables and RPE) when individuals were required to produce maximal motor output in VO_2MAX_-matched MIT and TT_4km_. Then, we were able to observe how the cerebral regulation responded to these different maximal aerobic exercise modes for the entire period of exercise.

Nevertheless, although these exercises have produced similar motor output (i.e., power output and EMG) during the last 50% of the trials, they were considerably different in duration (12 vs. 6 min). We are unaware if the different exercise durations could have affected our results in some way. For example, a recent study by (Thomas et al., 2015) found a reduced rest corticospinal excitability after 20 and 40 km self-paced cycling time trial, but not after TT_4km_. Accordingly, it was found different reductions in maximal voluntary activation after 4 km (↓ 6%), 20 km (↓ 12%), and 40 km (↓ 10%) cycling time trials. Thus, these results may have indicated that supraspinal and spinal sites (putative neuronal pathways along the corticospinal tract) responded differently between cycling trials of different durations, even though the different mean power output (i.e., motor output) between them may have further influenced the results. It is important to note that the direct comparison between studies should be made carefully, as we measured PMC activation through EEG technique during dynamic cycling, whereas that study used a maximal voluntary contraction superimposed by TMS, after the exercise cessation (Thomas et al., 2015). In this sense, rather than conflicting one with each other, these studies may indicate that cerebral regulation is complex, and differs between exercises different in nature (maximal dynamic cycling vs. maximal sustained contraction), as suggested elsewhere (Liu et al., 2005). Perhaps the combination of NIRS and EEG, together with maximal voluntary contraction superimposed by TMS, in different aerobic exercise modes of equivalent duration, such as conventional and perceptually-controlled MIT (Mauger and Sculthorpe, 2012), may respond how the different durations affected our results.

The use of EEG technique to monitor changes in cortical activation during whole body strenuous aerobic exercises has been criticized due to the inherent noise derived from upper body movement and low spatial resolution (Thompson et al., 2008; Enders and Nigg, 2016). Regarding the first aspect, we took some precaution in order to record good-quality EEG signal during exercise by using active electrodes, fixing cables and electrode, and asking individuals to maintain upper limbs as steady as possible during exercise (Thompson et al., 2008; Hilty et al., 2011). Furthermore, muscle artifacts seem to be more prevalent for frontal and posterior cortical sites than for Cz position, whereas eye movements may not increase during exercise to highly affect EEG signal, mainly the α bandwidth (Thompson et al., 2008; Hilty et al., 2011). However, we acknowledge that EEG artifacts associated with whole body strenuous exercises are a challenge to data analysis, being the reason why we actually excluded two individuals from the EEG analysis.

Regarding the low spatial resolution, it is not possible to ensure that we measured the exact cortical area responsible for the motor command of primary muscles involved in cycling (e.g., VL and RF muscles). We used the EEG international 10–20 system because we were interested in an overview of the cerebral responses during maximal cycling aerobic exercises; that is PFC oxygenation and PMC activation. Therefore, according to the Penfield's human motor Homunculus we have selected a cortical area that should satisfactorily reflect the motor command for lower limbs (hip, knee, ankle, and toes; Goodall et al., 2014), however we cannot ensure that we measured the exact area responsible for VL and RF muscles recruitment.

Although the limitations highlighted above may require some caution when interpreting cerebral responses to strenuous aerobic exercises, results of the present study may further suggest some practical implications. For example, it would be worth to know if long-term endurance training may modulate the individual's ability to tolerate PFC deoxygenation, while maintaining PMC activation. Perhaps, improvements in motor output performance after training period could be also related to a modulation of activation in these cerebral areas.

## Conclusion

The present study showed that similar, maximal motor output was attained at the closing stages of different VO_2MAX_-matched aerobic exercises, regardless of different cerebral responses during most of the exercises duration. The decrease in PFC oxygenation during the last percentages of the trials impaired neither motor output (EMG and power output) nor PMC activation, suggesting that the different COX responses during exercise may be part of a centrally-coordinated regulation.

## Author contributions

All of the listed authors contributed to this study, conceiving and designing the experiments (FP, TN, and CU), collecting and analyzing the data (FP, CD, RC, FP, FM, and CU), writing the manuscript (FP, FP, FM, and CU), criticizing and reviewing the manuscript (CD, RC, TN, and AG). All the listed authors approved the final version of the manuscript.

## Funding

The FAPESP-Brazil sponsored the first author (process 2010/01317-0) and CNPq-Brazil sponsored the last author of this study. This study was supported by FAPESP (#2005/56578-4 and #2015/13096-1) and CNPq-Brazil (480702/2010-1 and 303085/2015-0).

### Conflict of interest statement

The authors declare that the research was conducted in the absence of any commercial or financial relationships that could be construed as a potential conflict of interest.

## Abbreviations

COX: cerebral oxygenation
DHb: hemoglobin difference
EEG: electroencephalography
EMG: electromyography
NIRS: near-infrared spectroscopy
HHb: deoxy-hemoglobin
MIT: maximal incremental exercise test
MOX: muscular oxygenation
O_2_Hb: oxy-hemoglobin
PeTCO_2_: end-tidal carbon dioxide tension
PFC: prefrontal cortex
PMC: primary motor cortex
RF: rectus femoris
RMS: root mean square
RPE: ratings of perceived exertion
THb: total hemoglobin
TT_4km_: 4 km time trial
VE: ventilation
VL: vastus lateralis
VCO_2_: carbon dioxide production
VO_2_: oxygen uptake
VO_2MAX_: maximal oxygen uptake
W_PEAK_: peak power output.
